# A Novel Regulator Participating in Nitrogen Removal Process of *Bacillus subtilis* JD-014

**DOI:** 10.3390/ijms22126543

**Published:** 2021-06-18

**Authors:** Ting Yang, Yi Shi, Qian Yang, Yu Xin, Zhenghua Gu, Liang Zhang

**Affiliations:** 1 Key Laboratory of Industrial Biotechnology, Ministry of Education, Jiangnan University, Wuxi 214122, China; lalating2009@163.com (T.Y.); shiyi0621@jiangnan.edu.cn (Y.S.); yangqian9066@163.com (Q.Y.); yuxin@jiangnan.edu.cn (Y.X.); guzhenghua1975@163.com (Z.G.); 2 National Engineering Laboratory for Cereal Fermentation Technology, Jiangnan University, Wuxi 214122, China

**Keywords:** *Bacillus subtilis*, aerobic denitrification, transcriptome, nitrite reductase

## Abstract

Aerobic denitrification is considered as a promising biological method to eliminate the nitrate contaminants in waterbodies. However, the molecular mechanism of this process varies in different functional bacteria. In this study, the nitrogen removal characteristics for a newly isolated aerobic denitrifier *Bacillus subtilis* JD-014 were investigated, and the potential functional genes involved in the aerobic denitrification process were further screened through transcriptome analysis. JD-014 exhibited efficient denitrification performance when having sodium succinate as the carbon source with the range of nitrate concentration between 50 and 300 mg/L. Following the transcriptome data, most of the up-regulated differentially expressed genes (DEGs) were associated with cell motility, carbohydrate metabolism, and energy metabolism. Moreover, gene *nirsir* annotated as sulfite reductase was screened out and further identified as a regulator participating in the nitrogen removal process within JD-014. The findings in present study provide meaningful information in terms of a comprehensive understanding of genetic regulation of nitrogen metabolism, especially for *Bacillus* strains.

## 1. Introduction

The releasing of severe excessive amounts of nitrogenous substances into aqueous systems is one of the important reasons for the continuous degradation of the water quality in aqueous systems [1]. This pollutant mainly results from the improper disposal of industrial wastewater, excessive use of artificial fertilizers, and indiscriminate combustion of fossil fuel. As a result, the excessive accumulation of nitrate in waterbodies poses a potential threat to both aquatic life and human beings [2,3,4]. To eliminate the problem, various treatment methods such as filtration, ion exchange, physical adhesion, and electrodialysis have been implemented for the removal of nitrate contents before the influent of wastewater into aquatic systems [2]. Nevertheless, there are several limitations for the practical application of the above technologies, owing to the reality that most of these techniques are associated with high expensive operating costs, complicated operation process, high energy consumption, and the generation of secondary pollution [5,6].

In contrast, biological denitrification process is recognized as the most effective and eco-friendly strategy to remove the nitrate contaminants in wastewater. In particular, aerobic denitrifying bacteria, which could utilize nitrate and oxygen as electron acceptors at the same time and efficiently convert nitrate to harmless nitrogen gaseous under aerobic conditions, has demonstrated to be a promising alternative candidate for degrading nitrogen pollution [7,8,9]. To date, numerous microorganisms with aerobic denitrification performance have been isolated and characterized [10,11,12,13,14]. Among these functional strains, the denitrifying *Bacillus* species have shown great advantages in the application of bioremediation of polluted water, relying on their own characteristics, such as biosecurity, strong stress resistance, easy cultivation, convenient to transport, and long-term storage by the production of endospores [15,16,17].

*Bacillus subtilis* JD-014 is a wild strain that has been isolated and identified with the capability to remove nitrogen pollutants via aerobic denitrification process [18]. Based on previous studies related to similar functional strains, the type of carbon sources, as well as the initial concentration of nitrate were suggested to be important factors that would affect the biological nitrate removal performance and the regulation process of nitrogen metabolism [19]. Hence, to further verify the application potential of JD-014, evaluation is necessary regarding its denitrification performance against different carbon sources and initial nitrate concentrations.

Furthermore, due to the fact that there is limited research about the denitrification metabolic pathway in the genus *Bacillus*, no comprehensive knowledge could be applied to understand the regulation metabolism of nitrogen degradation in this species, especially under genus level. Therefore, to elucidate the aerobic denitrification pathway in *Bacillus* species, the complete genome of JD-014 was sequenced and some functional enzymes were annotated to the conventional denitrification process in the chromosome of JD-014 via homologous comparison [20]. However, it was noted that there was a lack of a functional gene annotated with nitrite reductase (Nir) in strain JD-014 based on the available genetic annotation information. According to previous molecular analysis for typical denitrifying bacteria, Nir is a key denitrifying enzyme that catalyzes the transform of NO_2_^−^ to NO [21]. The absence of Nir within JD-014 suggested that there should be an alternative enzyme to accomplish the denitrification process for the reason that excessive accumulation of nitrite within the cell would bring increasing biotoxicity to the bacterium itself [22]. In recent years, transcriptomic technology has been widely used and proved to be a useful tool for screening candidate biomarkers responding to different pollutants [23,24,25]. For strain JD-014, following the transcriptomic data, the potential genes involved in the aerobic denitrification metabolism process are supposed to be identified.

Considering all of the above, the effect of varied carbon sources and nitrate concentrations on the nitrogen removal performance of JD-014 was evaluated firstly, and the transcriptome corresponding to different nitrate concentrations was analyzed for the purpose to screen candidates related to aerobic denitrification. The overall aim is to provide novel molecular information for establishing the complete aerobic denitrification pathway in *Bacillus* species, and hence to promote the application potential for the bioremediation of nitrogen pollutants contaminated environments.

## 2. Results and Discussion

### 2.1. Effect of Carbon Sources and Nitrate Concentrations on Nitrogen Removal Performance of JD-014

**Figure 1:**
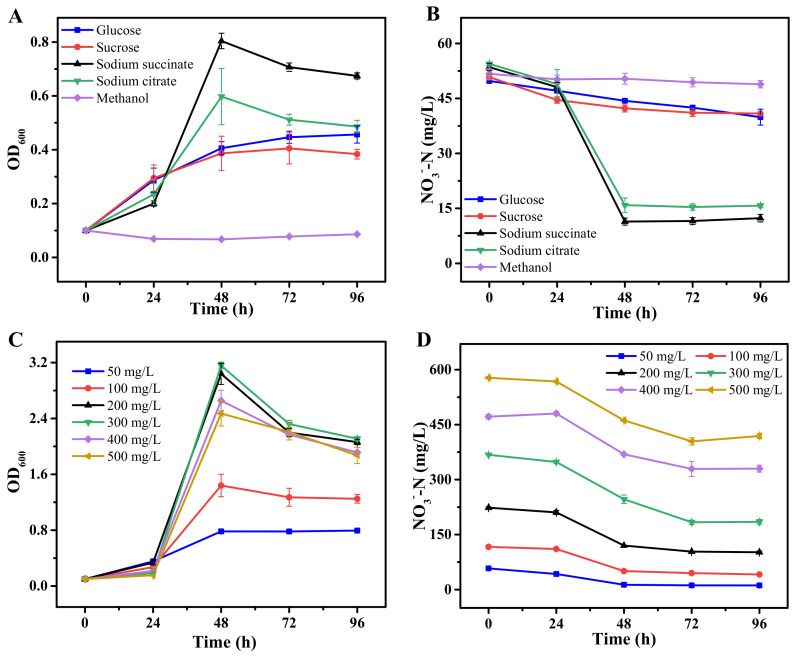
Effect of different carbon sources (**A**,**B**) and nitrate concentrations (**C**,**D**) on the growth and nitrogen removal performance of JD-014.

Figure 1 illustrates the response of strain JD-014 to different carbon sources and nitrate concentrations by comparing the nitrogen removal performance. It has been suggested that carbon source is an important factor affecting denitrification process, since different carbon sources would induce variations in electron transfer process and the associated activity of enzymatic reaction within heterotrophic denitrifiers [6,26]. The results showed in Figure 1A,B indicate that the cell growth and nitrogen removal performance of JD-014 were quite different among the five tested carbon sources. The highest nitrate removal rate was achieved when sodium succinate and sodium citrate were used as the sole carbon sources. Under such conditions, JD-014 could have 76.95% and 71.09% of the nitrate removed with the corresponding maximum cell density OD_600_ of 0.80 and 0.60, respectively. However, when the carbon source was changed to glucose or sucrose, only 19.84% and 19.56% of the nitrate were removed by JD-014, which is much lower than that with sodium succinate and sodium citrate. In addition, it was found that JD-014 could neither grow nor utilize nitrate when methanol was provided as the sole carbon source.

It has been reported that carbon source with a simple structure and low molecular weight might be more suitable for the growth of bacteria as well as for serving as electron donors during denitrification, as they could easily participate in simple biochemical pathways of carbon source utilization [27,28]. In agreement with previous studies [10,29], the current results also indicated that a higher NO_3_^−^-N removal efficiency of JD-014 was achieved when sodium succinate and sodium citrate were applied as the carbon source. This may because sodium succinate and sodium citrate could directly participate in the TCA cycle, making it easier to be efficiently utilized by denitrifiers. In comparation, macromolecular organic substances such as glucose and sucrose normally required to be hydrolyzed into small molecular organic acids before utilization by microorganisms [30,31,32]. Taking the growth rate and nitrate removal efficiency into account, it was suggested that strain JD-014 preferred sodium succinate mostly for grow and thereafter this carbon source was chosen for the subsequent experiment.

The effect of nitrate concentrations on cell growth and nitrogen removal performance were depicted in Figure 1C,D. A total of six different concentrations were investigated, and the results demonstrated that the growth rate increased with the increasing of NO_3_^−^-N concentrations between 50 and 300 mg/L. The corresponding maximum OD_600_ for these four incubation conditions were 0.79, 1.44, 3.04, and 3.15, respectively. While for nitrate removal efficiency, it was found that 80.28% of the nitrate could be removed when the initial nitrate concentration was 50 mg/L. With the increase of nitrogen concentration to 300 mg/L, the final removal efficiency declined to 49.76%. In comparison, although strain JD-014 could grow well with a maximum OD_600_ of 2.66 and 2.47 when increasing the initial concentration of nitrate to 400 and 500 mg/L, the final nitrate removal rate was only observed to be less than 30%. According to previous research, the threshold of denitrifying bacteria for nitrate concentration depended on the tolerance limit of microorganism itself and also the availability of carbon source [33]. Since the C/N ratio was maintained as 10 for all the cultural conditions in this study, the nitrate concentration of 300 mg/L was considered as the maximum nitrogen loading concentration for strain JD-014. In combination with a previous observation that the production of N_2_ during the nitrate removal process could be maintained at a relative high level (16.39–29.31 mg/L) when the concentration of nitrate was controlled as 50 mg/L [18], 50 and 300 mg/L were selected as the low and high concentration groups, respectively, to analyze the differentially expressed genes during the denitrification process in a further transcriptome study.

### 2.2. Transcriptomic Analysis of JD-014 during Denitrification Process

**Figure 2:**
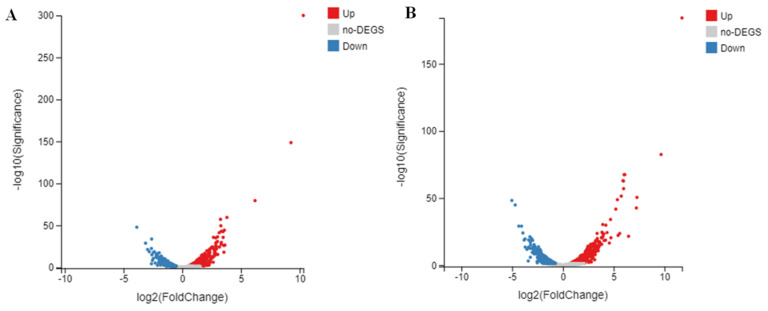
Volcano plot of DEGs between control and treated samples of YE50 (**A**) and YE300 (**B**) during denitrification process. Red dots represent up-regulated genes, blue dots represent down-regulated genes, and grey dots indicate genes without difference.

A transcriptome analysis was carried out to identify the differentially expressed genes (DEGs) associated with denitrification process. Compared with the control group, there were 567 DEGs in the treated sample YE50, of which 349 genes were up-regulated and 218 genes were down-regulated. While in the treated sample YE300, more DEGs were found, with 599 up-regulated genes and 447 down-regulated genes (Figure 2).

**Figure 3:**
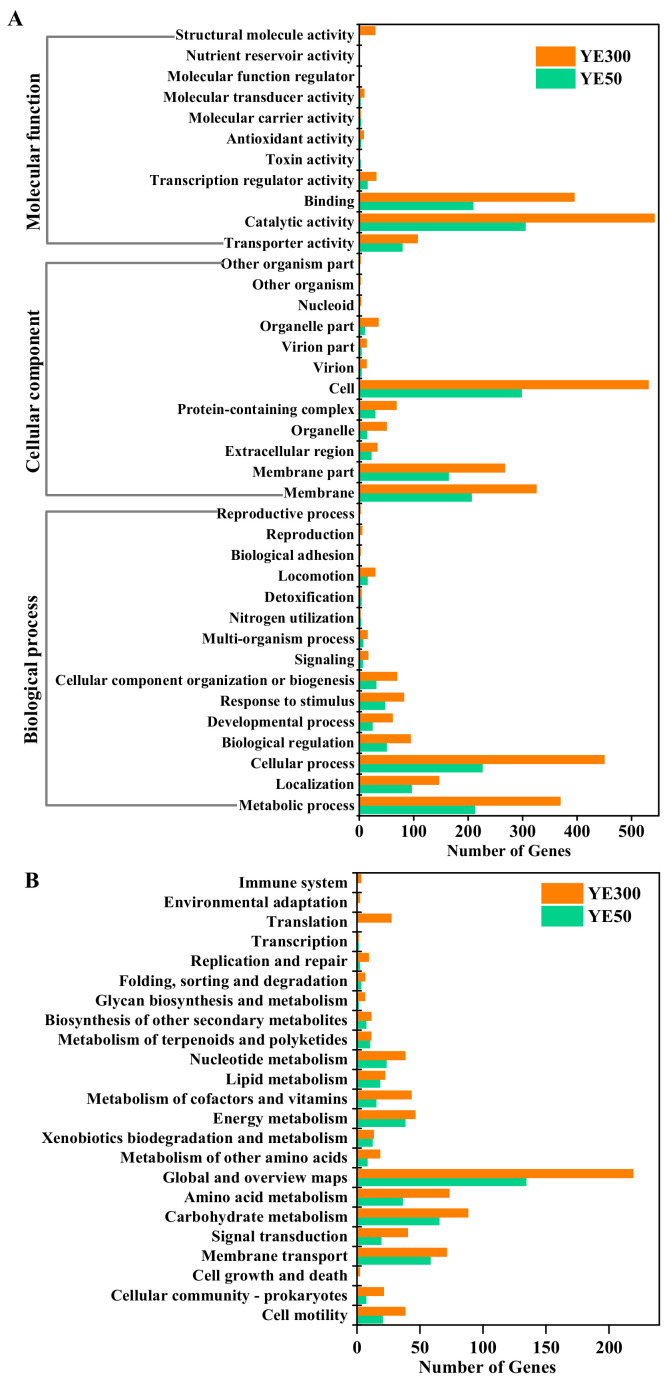
The Gene-Ontology terms (**A**) and the KEGG pathway (**B**) analysis of differential expressed genes (DEGs) for JD-014 against low and high concentrations of nitrate (YE50 and YE300) during denitrification process. The control was incubated within media without nitrate.

To comprehensively analyze the properties and specific function of these DEGs, GO and KEGG databases were applied for further annotation and categorization. Through GO analysis, a total of 498 DEGs in group YE50 and 921 DEGs in YE300 were mapped to 30 and 38 GO terms respectively in three main categories, including molecular function, cellular component, and biological process. Among these categories, it was noticed that most DEGs in YE50 and YE300 were associated with functions annotated as binding, catalytic activity, cell, cellular process, and metabolic process (Figure 3A).

The KEGG pathway analysis indicated that compared with the control, a total of 247 DEGs in group YE50 were assigned to 19 pathways, while in YE300, 430 DEGs were linked to 23 pathways (Figure 3B). Most of these DEGs in both groups that involved in cell motility, signal transduction, xenobiotics biodegradation and metabolism, and metabolism of terpenoids and polyketides, were up-regulated. Moreover, the KEGG enrichment analysis depicted that DEGs associated with the ABC transporters pathway in group YE50 and YE30 were significantly enriched (Figure S1). This pathway was suggested to be associated with material transport and other studies about petroleum hydrocarbon degradation by *Achromobacter* sp. HZ01 has also observed an enrichment of DEGs in the above category [23]. During the degradation of nitrogen pollutants by strain JD-014, some harmful intermediates such as nitrite could be produced. As a response, genes related to material transport might then be triggered to overcome the biotoxicity caused by the accumulation of a toxic metabolite within the strain JD-014.

### 2.3. qRT-PCR Verification of Selected Genes

**Figure 4:**
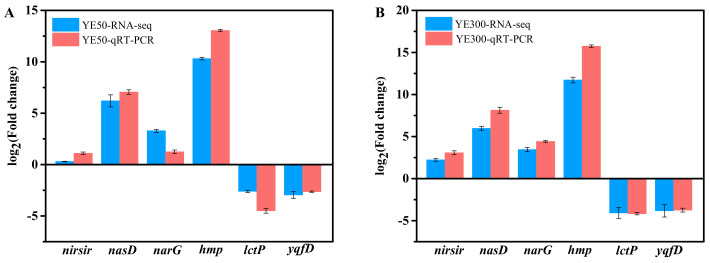
qRT-PCR validation of DEGs of JD-014 under the low concentrations of nitrate YE50 (**A**) and high concentrations of nitrate YE300 (**B**) compared to the control without nitrate during the denitrification process. Six randomly selected genes were *nirsir* (GE01386), *hmp* (GE03494), *nasD* (GE00279), *narG* (GE00997), *lctP* (GE00304), and *yqfD* (GE02214). The plus and minus values represent up-regulated genes and down-regulated genes, respectively.

The expression profile of DEGs obtained from transcriptome data was confirmed through six randomly selected DEGs using qRT-PCR analysis. Among these DEGs, *nirsir*, *nasD*, *narG,* and *hmp* were up-regulated in RNA-seq, while *lctP* and *yqfD* were down-regulated. The results shown in Figure 4 illustrated that there was a similar expression trend of these six DEGs between the RNA-seq and qRT-PCR analysis, indicating the reliability of the transcriptome results even though there was a minor variation of expression levels.

### 2.4. DEGs Related to Denitrification Process in JD-014

According to the overall analysis of DEGs associated with the *B. subtilis* JD-014 during nitrogen removal process, a large number of DEGs involved in various metabolic and regulatory processes, such as cell motility, carbohydrate metabolism, membrane transport, transcriptional regulators and energy metabolism, were found. Among these categories, cellular function of electron transport and consumption through the electron transfer chains (ETC) are considered to be essential for aerobic denitrification [34]. The canonical ETC system consists of NADH dehydrogenase (complex I), succinic dehydrogenase (complex II), quinone pool, the bc_1_ complex (complex III), cytochrome c, and terminal oxidase (complex IV) [35,36]. It was found that there were complete components of ETC systems in the chromosome of JD-014, and most of the key DEGs related to electron transport were up-regulated in YE50 and YE300 (Table S1). Since the biological denitrification process includes a series of electron donating process, the comprehensive ETC system identified within JD-014 was in agreement with previous observations of the high nitrogen removal performance by this strain [18]. With the participation of this system, the transport of an electron to denitrifying enzymes might be more efficient. A similar observation was also reported in previous study of *Paracoccus pantotrophus* NJUST38 [34], which was also identified as an aerobic denitrifying strain; DEGs related to the ETC system were up-regulated in the presence of nitrate and further suggested that the production, transport, and consumption of electrons would be enhanced during the nitrate removal process.

The transcriptional analysis of genes involved in the carbohydrate metabolism showed that the expression level of most genes in TCA cycle and oxidative phosphorylation, such as succinate dehydrogenase, ATP synthase, cytochrome c oxidase, and cytochrome aa_3_ oxidase were also up-regulated (Tables S2 and S3). Hence, the energy metabolism was improved with the addition of nitrate, which could induce more NADH and FADH_2_ as electron donors to support the denitrification process. Moreover, it was reported that DctA was associated with the transport of succinate [27]. Herein, compared to the control without nitrate, the current results depicted that the *dctA* (GE00156)-encoding C_4_-dicarboxylate transporter was up-regulated 3.37- and 11.06-fold, respectively, in YE50 and YE300, indicating that the carbon metabolism of JD-014 was promoted during the aerobic denitrification with sodium succinate as a carbon source.

In addition, the expression of a series of functional genes involved in different nitrogen metabolism pathways during aerobic denitrification was also analyzed. In general, the reduction of nitrate to nitrite in the anaerobic conditions is catalyzed by membrane-bound nitrate reductase (Nar) [21]. All the functional genes *narG*, *narH*, *narJ,* and *narI* that encode this enzyme were significantly up-regulated 4.87–11.59-fold and 4.28–10.93-fold, respectively, in YE50 and YE300 under aerobic conditions, indicating that these functional genes were also involved in aerobic denitrification. Instead, genes related to nitric oxide reductase and nitrous oxide reductase, such as *norD*, *norQ,* and *nosD*, were down-regulated, with a less significant difference of 1.10–1.57-fold and 1.03–1.30-fold, respectively in YE50 and YE300 (Table S4). 

Unlike conventional denitrifying bacteria, *nirS* or *nirK* genes, encoding for the nitrite reductase, which catalyzed the reduction of nitrite to nitric oxide in the denitrification process [21,37], were absent in the genome of JD-014. To figure out whether there were alternative genes related to nitrite reduction; gene *nirsir* annotated to encode sulfite reductase was screened out as the candidate through the transcriptional analysis in this study. As shown in Table S4, the expression of *nirsir* was increased with the rise of nitrate concentrations, which was up-regulated 1.23-fold in YE50, while in YE300 it was significantly up-regulated 4.68-fold. Based on the homology BLAST, the *nirsir* sequence showed 50.95–84.09% similarity to the *cysI* genes (encoding for the sulfite reductase) of different species, while it had relatively low similarities (<20% identity) to some well-known *nirS*/*nirK* (Table 1). Although *nirsir* was not annotated to typical nitrite reductase, this gene was still considered as a candidate involved in the aerobic denitrification metabolic process of JD-014. The above hypothesis was put forward for the reason that *nirsir* was found to contain 4Fe-4S clusters by sequence alignment, which was the same as the conventional *nir* in the denitrification process. Therefore, it is speculated that *nirsir* might be simultaneously involved in the detoxification of NO_2_^−^ or SO_3_^2−^.

### 2.5. Functional Analysis of Nirsir under Aerobic Denitrification

**Figure 5:**
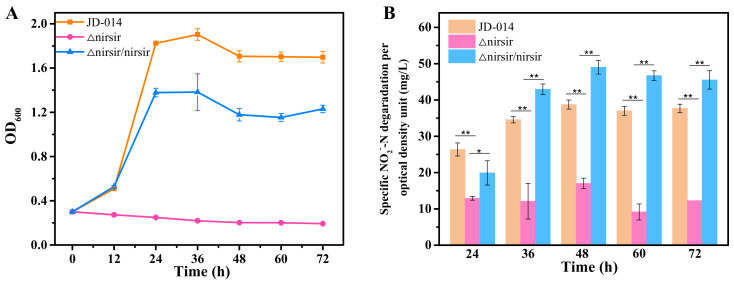
Comparison of nitrogen removal process among wild strain JD-014, *nirsir* knock-out mutant strain (Δnirsir), and the corresponding complementation strain (Δnirsir/nirsir). (**A**) The growth curves. (**B**) Nitrite removal characteristics. Statistical analysis was performed by using one-way analysis of variance (ANOVA) with Duncan’s test (* *p* < 0.05, ** *p* < 0.01).

**Figure 6:**
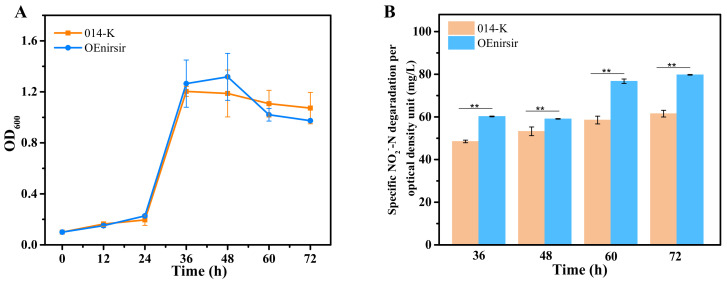
Comparison of nitrogen removal process between control strain (014-K) and *nirsir* over-expressing mutant strain (OEnirsir). (**A**) The growth curves. (**B**) Nitrite removal characteristics. Statistical analysis was performed by using one-way analysis of variance (ANOVA) with Duncan’s test (** *p* < 0.01).

To demonstrate the function of *nirsir* during the nitrogen removal process, knocked-out mutant (Δnirsir), retro-complementation mutant (Δnirsir/nirsir), and overexpression mutant (OEnirsir) strains were constructed. The growth and nitrogen removal performance of wild strain JD-014 and mutant strains were analyzed in the DM with NO_2_^−^-N as the sole N source. Since nitrite could cause toxicity to bacteria as well as inhibit the denitrification process [22], when the gene *nirsir* was knocked out, the mutant strain JD-014Δnirsir could not use nitrite as a nitrogen source to maintain growth, and also lost the ability to reduce NO_2_^−^-N (Figure 5). For the retro-complementation mutant (Δnirsir/nirsir), it was observed that the mutant could restore the partial cell growth and rescue the NO_2_^−^-N removal characteristic. When the gene *nirsir* was further overexpressed, it was found that the growth trend of the overexpression mutant OEnirsir was similar with that of the control (strain 014-K), which was the wild strain carrying the empty plasmid pMA5. However, the specific NO_2_^−^-N degradation per optical density of OEnirsir strain was higher than that of 014-K (Figure 6).

**Figure 7:**
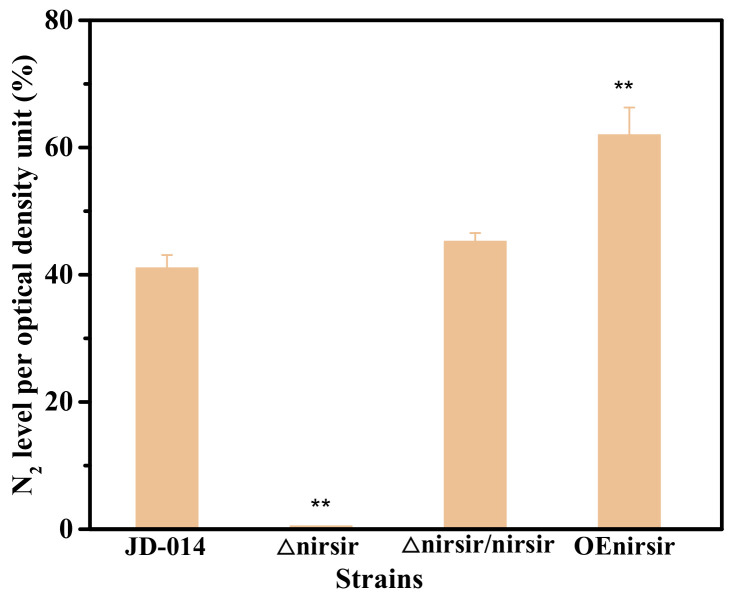
N_2_ level between wild strain JD-014 and mutant strains. Statistical analysis was performed by using one-way analysis of variance (ANOVA) with Duncan’s test (** *p* < 0.01).

The production of N_2_ for wild, knock-out mutant, retro-complementation mutant and the overexpressed strain was also measured. As shown in Figure 7, there was an observation of N_2_ accumulation for the above strains, except for knock-out mutant strain JD-014Δnirsir. After the complement of the *nirsir*, the retro-complementation mutant strain Δnirsir/nirsir recovered the ability of aerobic denitrification, and the production of N_2_ was basically the same as that of the wild strain JD-014. When the gene *nirsir* was further overexpressed in JD-014, the production of denitrifying product N_2_ was also enhanced. The above results demonstrated that *nirsir* may play an important role in the regulation of the nitrogen removal process within JD-014.

**Figure 8:**
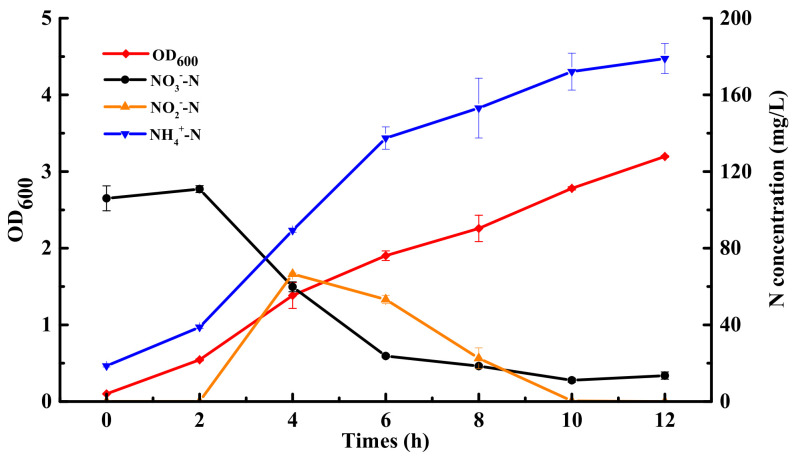
The nitrogen removal performance of the knock-out mutant strain JD-014Δnirsir on YDM.

Furthermore, to further identify whether *nirsir* participates in the dissimilatory or assimilatory nitrogen reduction in JD-014, the nitrogen removal characteristic of the knock-out mutant strain JD-014Δnirsir was investigated in the DM with NO_3_^−^-N as the sole N source. However, JD-014Δnirsir was observed without capability to remove NO_3_^−^-N from the culture or even use it for growth. Together with the observation in nitrite only incubation test, it was speculated that the *nirsir* might be an indispensable gene associated with the capability to utilize inorganic nitrogen for the growth in strain JD-014. This hypothesis was verified by having the mutant strain JD-014Δnirsir inoculated with YDM, in which JD-014Δnirsir could grow and degrade the NO_3_^−^-N with the supplement of organic nitrogen source (Figure 8). The nitrate removal efficiency could reach 87.24% at 12 h. During this process, there was an accumulation of nitrite with a peak at 4 h (66.56 mg/L) and then gradually degraded. The degradation of nitrite might have been contributed to by several processes, including its own oxidation and assimilatory reduction to ammonia by NirA. Hence, the mutant could still utilize the organic nitrogen source for growth and convert the intermediate nitrite into other nitrogenous substances despite the elimination of *nirsir*. Given all that, rather than only regulating nitrite reduction, the gene *nirsir* also plays an essential role in both the nitrogen assimilation and dissimilation pathways in strain JD-014. 

However, as there is no enough research about the molecular mechanism of aerobic denitrification in *Bacillus* species, the current database could only provide limited information for denitrifying related genes’ annotation. In addition, rather than encoding the conventional denitrifying enzymes, unknown genes coding enzymes or multi-functional enzymes might also exist in bacteria with the ability of aerobic denitrification. As described in recent reports, there was a specific group of bacteria lacking functional denitrification genes *nirK* or *nirS,* which are still capable of reducing NO_2_^−^-N to gaseous products [38,39]. In combination with the current observation, the existence of *nirsir* in strain JD-014 could provide a theoretical reference for further study about atypical denitrifying bacteria.

## 3. Materials and Methods

### 3.1. Bacterial Strain and Culture Medium

*Bacillus subtilis* JD-014 was a wild strain that had been isolated from aquaculture pond in our previous work and identified with efficient denitrification properties under aerobic conditions [18]. Luria Bertani (LB) medium was used for the primary culture of strain JD-014. The denitrification medium (DM) was prepared for nitrogen removal performance evaluation, and the modified denitrification medium (YDM) (DM with 5 g/L yeast extract) was used for transcriptomic analysis. DM was composed as follows (per liter): 4.0 g Na_2_HPO_4_, 1.5 g KH_2_PO_4_, 0.2 g MgSO_4_·7H_2_O, 2.81 g sodium succinate, and 2 mL trace element solution [18].

### 3.2. Carbon Sources and Nitrate Concentrations Influence on Denitrification of JD-014

The variation of denitrification performance of JD-014 was investigated against different carbon sources and nitrate concentrations. Cells of JD-014 in LB medium harvested at the logarithmic phase were inoculated in 100 mL DM with the single factor being adjusted according to the experimental design. To study the effects of carbon sources, glucose, sucrose, methanol, sodium succinate, and sodium citrate were separately used as the sole carbon source in the DM. While in nitrate concentrations experiments, initial NO_3_^−^-N were adjusted to 50, 100, 200, 300, 400, and 500 mg/L by altering the amount of sodium nitrate in the DM, with a constant C/N ratio of 10 by adjusting the content of sodium succinate. All the above experiments were repeated three times and incubated at 37 °C, 200 rpm for 96 h, with an initial OD_600_ at 0.1. Samples were taken regularly along the tests to measure the cell density (OD_600_) and the concentration of nitrate (NO_3_^−^-N).

### 3.3. Transcriptome Analysis

Cell suspension of JD-014 in YDM in the presence or absence of nitrate was cultured at 37 °C, 200 rpm, and the cells were harvested at 4 h to conduct the transcriptomic analysis. Cells in the YDM with 50 or 300 mg/L initial nitrate were set as treated sample (named as YE50 and YE300), while the culture grown in media without nitrate in YDM was set as the control group. All the samples, containing three biological replications, were harvested at 4 °C, 12,000 rpm for 10 min. The pellets were immediately frozen by liquid nitrogen and stored at −80 °C for RNA extraction, then transported to Beijing Genomics Institute (BGI) for transcriptome analysis. The sequencing was performed on the BGISEQ-500 platform (BGI Tech., Wuhan, China).

Clean reads were obtained by removing the raw reads with low quality, adaptor polluted, and ambiguous reads [40]. After filtration, high-quality data was mapped to the *Bacillus subtilis* JD-014 reference genome (GenBank accession code: CP045478-045479) using HISAT 2-2.1.0 [41] and Bowtie 2-2.2.5 [42]. The expression levels of genes and transcripts were calculated with RSEM 1.2.12 [43], and the differentially expressed genes (DEGs) were identified using DEseq2 [44]. The criteria for DEGs were assigned as |log_2_ (fold change)| ≥ 1 and adjusted *p* value < 0.05 [45]. Raw sequence data in three biological replicates were deposited in the NCBI Sequence Read Archive (SRA) database (accession number PRJNA692756).

### 3.4. Quantitative Real Time PCR

To validate the reliability of the transcriptome data, six DEGs were randomly selected for qRT-PCR analysis. The primers used in the experiment were listed in Table 2. Total RNA samples were in accordance with that used in transcriptome analysis, and reverse transcribed with random primers to cDNA by using HiScript^®^ II 1st strand cDNA synthesis kit (Vazyme, Nanjing, China). Obtained cDNA was used as a template for qRT-PCR, and the housekeeping gene gyrase B (*gyr* B) was chosen as the relative quantity reference. qRT-PCR was performed in triplicates using ChamQ™ universal SYBR^®^ qPCR master mix kit (Vazyme, Nanjing, China) on CFX96 Bio-Rad Real-Time PCR System. The relative gene expression results were calculated according to the 2^−ΔΔCt^ method [46].

### 3.5. Construction of Nirsir Knockout Mutant Strain of JD-014

The mutant JD-014Δnirsir strain was constructed using the homologous recombination knockout method [47]. The thermosensitive rolling-circle replication plasmid pNZT1 was used as the backbone to construct the knockout plasmid. The *nirsir* gene of JD-014 was amplified using primers nirsir-KpnI-F/nirsir-XhoI-R and the purified product was TA cloned into the vector pMD19-T. Obtained plasmid 19T-nirsir was linearized by inverse PCR amplification with primers FX-nirsir-NheI-F/FX-nirsir-BamHI-R. In addition, the tetracycline resistance gene with FRT site was amplified from plasmid pHY300-PLK using primers FRT-BamHI-Tet-F/FRT-PstI-Tet-R. The fragment was inserted into the linearized plasmid 19T-nirsir, resulting in the gene-knockout cassette (nirsir-FRT-Tet-FRT-nirsir). Subsequently, the gene-knockout cassette was cloned into pNZT using the KpnI/XhoI restriction sites and transformed into competent cells of *Escherichia coli* JM109. After selection of the positive clones in LB plates containing ampicillin, the recombined knockout plasmid pNZT1-tet-Δnirsir was transferred into JD-014 by electroporation. Then colonies with the integrated plasmid were picked up on LB tetracycline agar plates to perform two-step replacement recombination [47]. The deletion mutants were verified by PCR with primers of yz-nirsir-F/yz-nirsir-R and sequencing. All primers used were listed in Table 2.

### 3.6. Construction of Nirsir Complementation and Overexpression Mutant Strain of JD-014

For the construction of *nirsir* complementation and overexpression of mutant strains, a fragment of *nirsir* was amplified from JD-014 genomic DNA and then cloned into the vector pMA5 with the ClonExpress^®^ II one-step cloning kit (Vazyme, Nanjing, China). The recombinant plasmid pMA5-nirsir was confirmed by sequencing and transformed into competent cells of JM109 for overnight cultivation in LB ampicillin solid medium at 37 °C. Positive clones were subsequently transformed into JD-014 and the mutant JD-014Δnirsir strain respectively through electro transformation, and selected in LB plates with 30 μg/mL kanamycin at 37 °C for 12 h. The complementation and overexpression mutants were verified by PCR with primers of pMA5-a-F/pMA5-a-R.

### 3.7. Analytical Methods

The cell density was measured at 600 nm using a spectrophotometer. Nitrate nitrogen (NO_3_^−^-N) and nitrite nitrogen (NO_2_^−^-N) were determined according to the standard method [48]. The concentration of N_2_ was detected by gas chromatograph with thermal conductivity detector (TCD) [18]. The nitrogen removal efficiency was calculated as (T_1_−T_2_)/T_1_ * 100%, where T_1_ and T_2_ represent the initial concentration of nitrogen and nitrogen concentration at time T, respectively. The statistical differences were examined by one-way analysis of variance (ANOVA) with Duncan’s test. *p* value < 0.05 (*) was considered to show significant difference and *p* value < 0.01 (**) as extreme difference.

## 4. Conclusions

In this study, the nitrogen removal performance of an isolated aerobic denitrifying strain, *Bacillus subtilis* JD-014, was characterized against different carbon sources and nitrate concentrations. It was found that JD-014 could degrade nitrate pollution efficiently with sodium succinate as a carbon source within the range of nitrate concentration of 50–300 mg/L. The transcriptome analysis was further conducted for better understanding the metabolic pathway associated with aerobic denitrification. The results revealed that the differentially expressed genes were mainly involved in the carbohydrate metabolism, energy metabolism, membrane transport, and transcriptional regulators. In particular, a novel candidate gene *nirsir* related to nitrite reduction was screened out from the transcriptional data and was verified with the function relating to the regulation of nitrogen removal process in JD-014. Overall, the study provided meaningful theoretical information for in-depth understanding of the molecular mechanism of nitrogen removal in *Bacillus* genus.

## Figures and Tables

**Table 1:** Comparison of *nirsir* with *cysI* and *nirS*/*nirK* in classical denitrification.

Genes	Description	Strains	Size (aa)	Identity (%)	Cover (%)	Accession Number
*nirsir*	sulfite reductase subunit beta	*Bacillus subtilis* JD-014	571	-	-	in CP045478
*cysI*	sulfite reductase [NADPH] hemoprotein beta-component	*Bacillus* sp. URHB0009	572	84.09	99	WP027319807
		*Mesorhizobium* sp. Root172	573	50.95	97	WP056566970
		*Geobacillus* sp. BCO2	349	68.77	52	KPD00931
		*Paenibacillus* sp. P1XP2	517	58.99	86	KHF35799
		*Verrucomicrobiales*	394	54.06	67	MAJ16745
*nirS* (partial)	cytochrome cd_1_ nitrite reductase	*Bacillus cereus* GS-5	136	13.73	8	APM87481
		*Pseudomonas aeruginosa* CCUG 241	259	10.99	6	AAD26540
		Uncultured bacterium wA20	265	11.48	17	CAB76794
		*Pseudomonas* sp. R125	154	17.82	10	CAF25139
*nirK*	copper-type nitrite reductase	*Bacillus azotoformans* LMG 9581	353	13.41	13	EKN68572
		*Bacillus firmus* GY-49	353	10.66	30	AMQ34899
		*Nitrosococcus oceani* NS58	372	13.74	20	CCA61349
		Anammox organism KSU-1	337	11.56	21	GAB64238

**Table 2:** Primers used in this study.

Primer	Primer Sequences (5′-3′)
hmp-F1-RT	CAAACAGCCTGAACGGCAAA
hmp-R1-RT	CGGCTCGCGATACACAAATG
nasD-F1-RT	AAAGAAGCCATTTGCGGCTG
nasD-R1-RT	TTCCAGCCGAGCACATTCAT
narG-F1-RT	CTGGTTCAACTCCGACACGA
narG-R1-RT	AATCGTCTGCCACCCTTCAG
nirsir-F1-RT	AGCACTTTTGGATACGATCGCAGC
nirsir-R1-RT	TTCATGATATGCTCTCGTCCGCG
lctP-F1-RT	GATTGGCGTGTTCATCACCG
lctP-R1-RT	CAGCAAATCTGAACCCGCAC
yqfD-F1-RT	TGACAGTCCCGCTTGAAACA
yqfD-R1-RT	CCCAGATCGGGATTGCCAAA
gyrB-F1-RT	AAGCTGGGCAACTCAGAAGCACGG
gyrB-R1-RT	AGCCATTCTTGCTCTTGCCGCC
nirsir-KpnI-F	CGGGGTACCATGGTGACCAAAATTCTAAAAGCACCG
nirsir-XhoI-R	CCGCTCGAGTCGTACCGTCAGTTGTTGCTT
FX-nirsir-NheI-F	CTAGCTAGCGGCTGACAGCCAATCAGAACTT
FX-nirsir-BamHI-R	CGCGGATCCACCACTCGTATACCTCTGAGTGGA
FRT-BamHI-Tet-F	ACGGGATCCGAAGTTCCTATTCCGAAGTTCCTATTCTCTAG
	AAAGTATAGGAACTTCGGATCAATTTTGAACTCTCTCC
FRT-PstI-Tet-R	AACTGCAGGAAGTTCCTATACTTTCTAGAGAATAGGAACTT
	CGGAATAGGAACTTCGGGCCATATTGTTGTATAAG
yz-nirsir-F	GCATGACGTCCATAACACATTGCT
yz-nirsir-R	AGGAGTTCTCTCCACACTTGTCT
pMA5-a-F	GGAGCGATTTACATATGAGTTATGCAG
pMA5-a-R	ATCAGCTTGCTTTCGAGGTGAATTTCGA

## Supplementary Materials

The following are available online at https://www.mdpi.com/article/10.3390/ijms22126543/s1.
