# Supplementary material for: A Novel Regulator Participating in Nitrogen Removal Process of Bacillus subtilis JD-014
Source: Int J Mol Sci. 2021 Jun 18;22(12):6543. doi: 10.3390/ijms22126543 (PMC8234713; doi:10.3390/ijms22126543)
Supplement: Supplementary file 1 [file ijms-22-06543-s001.zip › ijms-1216808-supplementary.pdf]

Supplementary materials

Article

A novel nitrite reductase in aerobic denitrification process of *Bacillus subtilis* JD-014

Ting Yang [1,2], Yi Shi [1,2], Qian Yang [1,2], Yu Xin [1,2], Zhenghua Gu [1,2] and Liang Zhang [1,2,*]

[1] Key Laboratory of Industrial Biotechnology, Ministry of Education, Jiangnan University, Wuxi 214122, China; lalating2009@163.com (T.Y.); shiyi0621@jiangnan.edu.cn (Y.S.); yangqian9066@163.com (Q.Y.); yuxin@jiangnan.edu.cn (Y.X.); 327984744@qq.com (Z.H.); zhangl@jiangnan.edu.cn (L.Z.)

[2] National Engineering Laboratory for Cereal Fermentation Technology, Jiangnan University, Wuxi 214122, China

* Correspondence: zhangl@jiangnan.edu.cn; Tel.: +86-510-85918235

Supplementary Figure and Table legend

Figure S1. KEGG enrichment map of DEGs between control and treated samples of YE50 (**A**) and YE300 (**B**), respectively.

Table S1. DEGs involved in the ETC system.

Table S2. DEGs related to the TCA cycle.

Table S3. DEGs related to the oxidative phosphorylation.

Table S4. Potential genes that associated with denitrification.

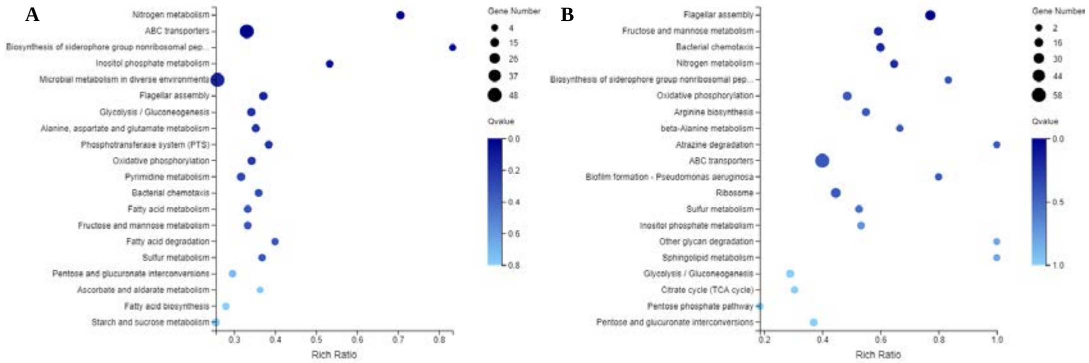

Figure S1. KEGG enrichment map of DEGs between control and treated samples of YE50 (**A**) and YE300 (**B**), respectively.

Table S1. DEGs involved in the ETC system.

Gene ID	Length	Description	$\log_2$(YE50/CK)	$\log_2$(YE300/CK)
complex I				
GE01572	1491	NADH-Ubiquinone/plastoquinone	1.46	1.49
GE02331	1149	NADH-dependent flavin oxidoreductase	-1.36	-2.21
GE03576	1179	NADH dehydrogenase-like protein *yjlD*	NA	-1.30
complex II				
GE01898	762	succinate dehydrogenase iron-sulfur subunit	1.01	1.04
complex III				
GE02502	768	menaquinol-cytochrome c reductase iron-sulfur subunit	1.14	NA
complex IV				
GE03296	1869	cytochrome c oxidase subunit I	1.85	2.57
GE03297	1071	cytochrome c oxidase subunit II	1.09	1.24
GE03295	624	cytochrome c oxidase subunit III	1.86	2.68
GE03294	333	cytochrome c oxidase, subunit IVB	1.69	2.83
GE03299	921	cytochrome oxidase assembly protein	NA	1.30
GE00848	1407	cytochrome bd ubiquinol oxidase subunit I	-1.05	-3.38
GE00849	1017	cytochrome bd ubiquinol oxidase subunit II	-0.77	-2.11
GE00909	1950	cytochrome aa_3 quinol oxidase, subunit I	1.99	NA
GE00910	615	cytochrome aa_3 quinol oxidase, subunit III	2.78	1.94
GE00911	375	cytochrome aa_3 quinol oxidase, subunit IV	2.73	2.02
GE01202	339	cytochrome c oxidase, cbb_3-type, subunit III	NA	1.23
GE03293	894	cytochrome c oxidase caa_3 assembly factor	1.05	2.37
others				
GE01888	774	electron transfer flavoprotein subunit beta	1.64	1.62
GE01889	978	electron transfer flavoprotein subunit alpha	1.67	1.60
GE03370	456	flavodoxin	2.79	3.12

"NA" indicates not DEGs in this group.

Table S2. DEGs related to the TCA cycle.

Gene ID	Length	Description	log_2(YE50/CK)	log_2(YE300/CK)
GE01827	1119	citrate synthase	NA	2.72
GE01828	1272	isocitrate dehydrogenase	NA	1.39
GE01829	939	malate dehydrogenase	NA	2.36
GE01898	762	succinate dehydrogenase/fumarate reductase	1.01	1.04
GE02978	2730	aconitate hydratase	NA	2.68
GE03325	1413	dihydrolipoamide dehydrogenase	1.49	1.46
GE03326	1329	pyruvate dehydrogenase E2 component	NA	1.62

"NA" indicates not DEGs in this group.

Table S3. DEGs related to the oxidative phosphorylation.

Gene ID	Length	Description	log_2(YE50/CK)	log_2(YE300/CK)
GE00424	1518	NADH dehydrogenase subunit 5	NA	-1.07
GE00848	1407	cytochrome bd ubiquinol oxidase subunit I	-1.05	-3.38
GE00849	1017	cytochrome bd ubiquinol oxidase subunit II	-0.77	-2.11
GE00909	1950	cytochrome aa_3 quinol oxidase, subunit I	1.99	NA
GE00910	615	cytochrome aa_3 quinol oxidase, subunit III	2.78	1.94
GE00911	375	cytochrome aa_3 quinol oxidase, subunit IV	2.73	2.02
GE01043	864	F_0F_1 ATP synthase subunit gamma	0.83	1.58
GE01044	1422	F_0F_1 ATP synthase subunit beta	1.73	1.95
GE01045	399	F_0F_1 ATP synthase subunit epsilon	1.72	1.85
GE01232	651	pyrophosphatase	NA	1.12
GE01898	762	succinate dehydrogenase / fumarate reductase	1.01	1.04
GE02502	768	menaquinol-cytochrome c reductase iron-sulfur subunit	1.14	NA
GE03294	333	cytochrome c oxidase, subunit IVB	1.69	2.83
GE03295	624	cytochrome c oxidase subunit III	1.86	2.68
GE03296	1869	cytochrome c oxidase subunit I	1.85	2.57
GE03297	1071	cytochrome c oxidase subunit II	1.09	1.24
GE03299	921	cytochrome oxidase assembly protein	NA	1.30
GE03576	1179	NADH dehydrogenase-like protein *yjlD*	NA	-1.30
GE03598	963	protoheme IX farnesyltransferase 1	NA	2.59

"NA" indicates not DEGs in this group.

Table S4. Potential genes that associated with denitrification.

Gene ID	Size (bp)	Description	log_2(YE50 /CK)	log_2(YE300/ CK)
GE00997	3687	nitrate reductase alpha subunit *narG*	3.29	3.45
GE00998	1464	nitrate reductase beta subunit *narH*	3.54	3.34
GE00999	555	nitrate reductase molybdenum cofactor assembly chaperone *narJ*	3.17	2.95
GE01000	672	nitrate reductase gamma subunit *narI*	2.28	2.10
GE02828	891	putative nitric oxide reductase *norQ*	-0.65	-0.33
GE02829	1917	putative activator of nitric oxide reductase *norD*	-0.14	0.04
GE00239	1755	putative nitrous oxide reductase family maturation protein *nosD1*	-0.28	-0.38
GE02717	1761	putative nitrous oxide reductase family maturation protein *nosD2*	0.25	0.62
GE01390	684	molybdenum ABC transporter permease *yvgM*	1.32	0.21
GE01391	783	molybdate ABC transporter substrate-binding protein *yvgL*	1.54	0.10
GE00277	2316	assimilatory nitrate reductase electron transfer subunit *nasB*	1.30	2.19
GE00278	2133	assimilatory nitrate reductase catalytic subunit *nasC*	1.01	1.64
GE00279	2418	nitrite reductase (NADH) large subunit *nirB*	6.20	5.97
GE00280	321	nitrite reductase (NADH) small subunit *nirD*	3.80	3.84
GE01386	1716	putative nitrite reductase *nirsir*	0.30	2.23
GE00179	498	nitrate/nitrite response regulator protein *narP*	0.98	-0.23
GE03040	408	transcriptional regulator protein *glnR*	1.08	1.37
GE01074	351	nitrogen regulatory PII-like protein *glnK*	0.84	0.09
GE00993	1188	nitrate/nitrite transporter *narK1*	3.48	3.94
GE00276	1206	nitrate/nitrite transporter *narK2*	1.05	1.24
GE02025	801	nitrite transporter *nirC1*	-0.43	-0.34
GE00919	771	nitrite transporter *nirC2*	0.10	-1.53
GE01075	1215	ammonium transporter *amt*	1.43	2.62
GE03040	408	MerR family transcriptional regulator *glnR*	1.08	1.37
GE00994	717	Crp/Fnr family transcriptional regulator *fnr*	2.27	2.90
GE00996	477	Crp/Fnr family transcriptional regulator *arfM*	1.84	1.73
